# INTEREST GROUP SESSION—AGING VETERANS: EFFECTS OF MILITARY SERVICE ACROSS THE LIFE COURSE: OPTIMIZING VETERAN AND CAREGIVER WELL-BEING THROUGH TECHNOLOGY

**DOI:** 10.1093/geroni/igz038.2071

**Published:** 2019-11-08

**Authors:** Christine E Gould, Julia Loup, Laura O Wray

**Affiliations:** 1 VA Palo Alto Health Care System, Palo Alto, United States; 2 University of Alabama, Department of Psychology, Tuscaloosa, Alabama, United States; 3 VA Center for Integrated Healthcare, Buffalo NY, United States

## Abstract

Older Veterans account for more than half of Veterans receiving care through the VA. Many suffer from dementia, traumatic brain injuries, and mental health disorders, resulting in complex care needs, which may include need for caregiver support. Innovative approaches using technology have the potential to improve access to treatments that improve the well-being of older Veterans and their caregivers. This symposium will present data from three Veterans Health Administrative investigators who are examining the impacts of innovative technology-delivered interventions. First, Dr. Michelle Hilgeman will describe preliminary 6-month outcome findings from a telephone-delivered Care Consultation + Counseling (CC+C) intervention for Veterans with dementia and their caregivers. Second, Dr. Kaci Fairchild will detail the background, methodology, and preliminary results of The COACH Project (Combined Online Assistance for Caregiver Health). The COACH Project is a mobile-delivered intervention consisting of exercise and caregiver skills training designed to reduce caregiver burden in those caring for someone with cognitive impairment. Third, Dr. Christine Gould will describe the development and feasibility of the Geri-Mobile Health clinic, which helps older Veterans use VA mental health apps to meet their well-being and mental health goals. Dr. Gould will describe the clinic’s personalized coaching method to support Veterans unfamiliar with apps and will share findings regarding the program’s acceptance, feasibility, and preliminary outcomes. Dr. Laura Wray, the executive director of the VA Center for Integrated Healthcare, will serve as the discussant. She will comment on implementing these interventions in primary care settings and the potential barriers to their adoption.

